# A prediction model of superimposed preeclampsia in women with chronic hypertension

**DOI:** 10.3389/fcvm.2025.1641662

**Published:** 2025-09-04

**Authors:** Yongjun Lu, Li Yang, Xiaoyan Li, Dan Kuai, Wenyan Tian, Huiying Zhang

**Affiliations:** ^1^ Department of Gynecology and Obstetrics, Tianjin Medical University General Hospital, Tianjin, China; ^2^ Tianjin Key Laboratory of Female Reproductive Health and Eugenics, Tianjin Medical University General Hospital, Tianjin, China; ^3^ Department of Obstetrics and Gynecology, Tongzhou Maternal & Child Health Care Hospital of Beijing, Beijing, China

**Keywords:** chronic hypertension, preeclampsia, direct bilirubin, cystatin-C, pre-pregnancy body mass index

## Abstract

**Objective:**

The incidence of chronic hypertension (CH) with superimposed preeclampsia (PE) is 5–6 times higher than that in non-CH pregnancies, with earlier onset, higher rates of multi-organ dysfunction, and poorer maternal/fetal outcomes, yet lacks dedicated screening tools. The purpose of this study is to establish and validate a prediction model for CH with superimposed PE.

**Methods:**

A retrospective case-control study was conducted on 471 CH patients admitted to the Tongzhou Maternal and Child Health Care Hospital of Beijing from January 2020 to December 2023. The patients were divided into superimposed preeclampsia (SPE) group (161 cases) and non-preeclampsia (NPE) group (310 cases) based on whether they had complicated PE. The patients were randomly divided into training set and validation set in a 7:3 ratio. General and clinical data were collected, and single-and multi-factor logistic regression analysis were used to screen for independent factors affecting PE in the training set. Based on the screening results, the diagnostic efficacy of PE was evaluated using the receiver operating characteristic curve. Risk prediction nomogram model was constructed using R language. The Bootstrap method was used to validate and produce calibration plots; the decision curve analysis (DCA) was used to assess the clinical benefit rate of the model.

**Results:**

The results of single factor and further multi-factor analysis showed that peripheral blood levels of direct bilirubin (DBIL), gamma-glutamyl transferase (GGT), glucose (GLU), cystatin-C (CysC) and pre-pregnancy body mass index (BMI) were independent influences on the occurrence of PE (*P* < 0.05). The area under the curve of the combined of DBIL, GGT, GLU, CysC and pre-pregnancy BMI was 0.789, with a sensitivity of 0.593 and a specificity of 0.830, which is better than a single clinical diagnostic indicator. The results of multifactor analysis were constructed as a nomogram model, and the mean absolute error of the calibration curve of the modeling set was 0.035, suggesting that the predictive probability of the model was generally compatible with the actual value. DCA showed the predictive model has clinical utility value.

**Conclusions:**

The occurrence of PE in women with CH is related to the peripheral blood levels of DBIL, GGT, GLU, CysC and pre-pregnancy BMI, and the combination of these indexes has a better clinical diagnostic value than a single index. The nomogram model constructed by using the above indicators can be used for the prediction of PE and has high predictive efficacy.

## Introduction

The incidence of chronic hypertension (CH) complicating pregnancy is 1%–5%, showing an increasing trend in recent years (1). The incidence of CH with superimposed preeclampsia (PE) is 5–6 times higher than that in non-CH pregnancies, and CH with superimposed PE is associated with earlier delivery (mean gestation: 33.9 weeks), 40% risk of fetal growth restriction, and 3-fold higher maternal mortality vs. *de novo* PE (2, 3). As a pregnancy-specific disease, PE has a global incidence of 2%–4%, causing approximately 46,000 maternal deaths and about 500,000 fetal and neonatal deaths annually worldwide (4).

The primary manifestations of PE are hypertension, often accompanied by proteinuria. Severe cases can lead to systemic organ dysfunction involving the heart, liver, lungs, kidneys, and nervous system, as well as fetal growth restriction, intrauterine fetal death, and maternal mortality (5). Due to the complex etiology and pathogenesis of PE, effective and highly specific predictive methods remain elusive. Research focuses on maternal factors, biomarkers, and biophysical parameters for PE prediction. Current biomarkers (e.g., sFlt-1/PlGF) are costly and rarely validated in CH with superimposed PE cohorts. Consequently, there is an urgent clinical need for predictive methods with superior performance, wider applicability, and lower economic costs.

The purpose of this study is to screen for risk factors of PE by retrospectively analyzing maternal factors, medical history, routine laboratory indicators, and factors occurring during pregnancy in pregnant women with CH complicating pregnancy. We aim to establish and validate a predictive model for CH with superimposed PE, providing a theoretical basis for early clinical identification and timely intervention.

## Materials and methods

### Study population

A retrospective case-control study was conducted on CH patients admitted to the Tongzhou Maternal and Child Health Care Hospital of Beijing from January 2020 to December 2023. Based on the presence or absence of PE, they were divided into a superimposed PE (SPE) group and a non-PE (NPE) group. The patients were randomly allocated into training and validation sets at a 7:3 ratio. Randomization was performed using SPSS 19.0's random number generator (seed = 2,000,000). At the same time, considering the incidence rate of superimposed PE (approximately 34.2%), a stratified sampling strategy was adopted, using “whether superimposed PE occurred” as the stratification variable, ensuring that the proportion of the two groups in the training set (NPE 217 cases, SPE 113 cases) and validation set (NPE 93 cases, SPE 48 cases) remained consistent with the total dataset (NPE 310 cases, SPE 161 cases), to avoid sampling bias affecting model stability. The training set was used for model construction, while the validation set was utilized for external validation.

### Inclusion criteria

1. Age 18–45 years old; 2. no smoking and intemperance; 3. single pregnancy. 4. Patients with CH and CH with superimposed PE, who delivered in the Tongzhou Maternal and Child Health Care Hospital of Beijing.

### Exclusion criteria

1. The diagnosis basis of CH and CH with superimposed PE in medical history data is insufficient; 2. other chronic kidney diseases, such as nephrotic syndrome, chronic nephritis, and other diseases that can cause changes in kidney function, such as systemic lupus erythematosus, allergic nephritis purpura; 3. various secondary hypertension, such as renal parenchymatous hypertension, renal vascular hypertension, primary hyperaldosteronism, neurocytoma, hypercortisolism, aortic coarctation; 4. multiple pregnancy; 5. cases with serious lack of clinical data.

### Grouping criteria

According to the Guidelines for the Diagnosis and Treatment of Hypertensive Disorders in Pregnancy (2020version) (6):

(1) NPE group–CH combined with pregnancy (non-PE): pregnant women with previous essential hypertension or systolic blood pressure ≥140 mmHg and/or diastolic blood pressure ≥90 mmHg before 20 weeks of pregnancy had no significant aggravation during pregnancy.
(2) SPE group–CH with superimposed PE: CH with new-onset proteinuria or other signs/symptoms of PE after 20 weeks or chronic proteinuria with new-onset hypertension.

### Research methods

#### General information collection

All pregnant women register at our hospital, undergo regular prenatal check-ups, and give birth there. Depending on their blood pressure, they receive either outpatient or inpatient treatment as needed. General information of patients, including age, height, pre-pregnancy weight, primiparity status, pregnancy method (natural conception/*in vitro* fertilization embryo transfer), past medical history [CH, diabetes, polycystic ovary syndrome (PCOS), PE, family history of hypertension], aspirin use (including gestational weeks of administration), antihypertensive medication use, number of prenatal visits, gestational weight gain, gestational hyperglycemia [including pre-existing diabetes, impaired fasting glucose, gestational diabetes mellitus (GDM), and whether pharmacological treatment was required]. Pre-pregnancy body mass index (BMI) should be calculated as: pre-pregnancy BMI = pre-pregnancy weight (kg)/[height (m)]^2^.

#### Blood pressure

Basic blood pressure including systolic blood pressure (SBP) and diastolic blood pressure (DBP), were collected. Patients were seated for 10 min, and SBP, DBP were measured using HBP-9020 blood pressure monitor (Omron, Dalian, China) by professional healthcare personnel.

#### Biochemical parameter detection

After 8 h of fasting, venous blood was collected in the morning from all participants in the first trimester of pregnancy (8–13 weeks), including hemoglobin (HGB), hematocrit (HCT), platelet (PLT), mean erythrocyte hemoglobin concentration (MCHC), platelet distribution width (PDW), prothrombin time (PT), prothrombin time activity (PTA), activated partial thromboplastin time (APTT), fibrinogen (FIB), thrombin time (TT), d-dimer (D-D), alanine aminotransferase (ALT), aspartate aminotransferase (AST), albumin (ALB), globulin (GLOB), total bilirubin (TBIL), direct bilirubin (DBIL), alkaline phosphatase (ALP), gamma-glutamyl transferase (GGT), glucose (GLU), blood urea nitrogen (BUN), creatinine (CREA), uric acid (UA), calcium (CA), total cholesterol (TC), triglyceride (TG), high density lipoprotein cholesterol (HDL-C), low density lipoprotein cholesterol (LDL-C), creatine kinase (CK), lactate dehydrogenase (LDH), creatine kinase isoenzyme (CK-MB), total bile acids (TBA), homocysteine (HCY), glycated albumin (GA), and cystatin-C (CysC).

### Statistical analysis

SPSS 19.0 (IBM, Armonk, NY, USA) and R language (R 4.3.3) software were used for statistical analysis. Normally distributed continuous variables were expressed as mean ± standard deviation and intergroup comparisons were performed using independent sample *t*-test. Non-normally distributed continuous variables are expressed as median (interquartile range) and intergroup comparisons were performed using Mann–Whitney *U* test. Categorical data are expressed as frequency and percentage and intergroup comparisons were performed using chi-square test. *P* values less than 0.05 were considered statistically significant. Single-and multiple-factor logistic regression analyses were used to screen for independent factors affecting PE in the training set. Based on the screening results, the diagnostic efficacy of PE was evaluated using the receiver operating characteristic (ROC) curve. ROC curves were plotted based on the predicted probabilities, and the area under the curve (AUC) was calculated. The optimal cutoff value was determined by Youden index (sensitivity + specificity − 1). R language (R 4.3.3) software was used to visualize the results of the multifactorial analysis to obtain the nomogram model and the computer simulation of the repeated sampling method (Bootstrap) was used to evaluate the calibration of the model; the decision curve analysis (DCA) was used to assess the clinical benefit rate of the model.

## Results

### Inclusion of the study population

**Figure 1:**
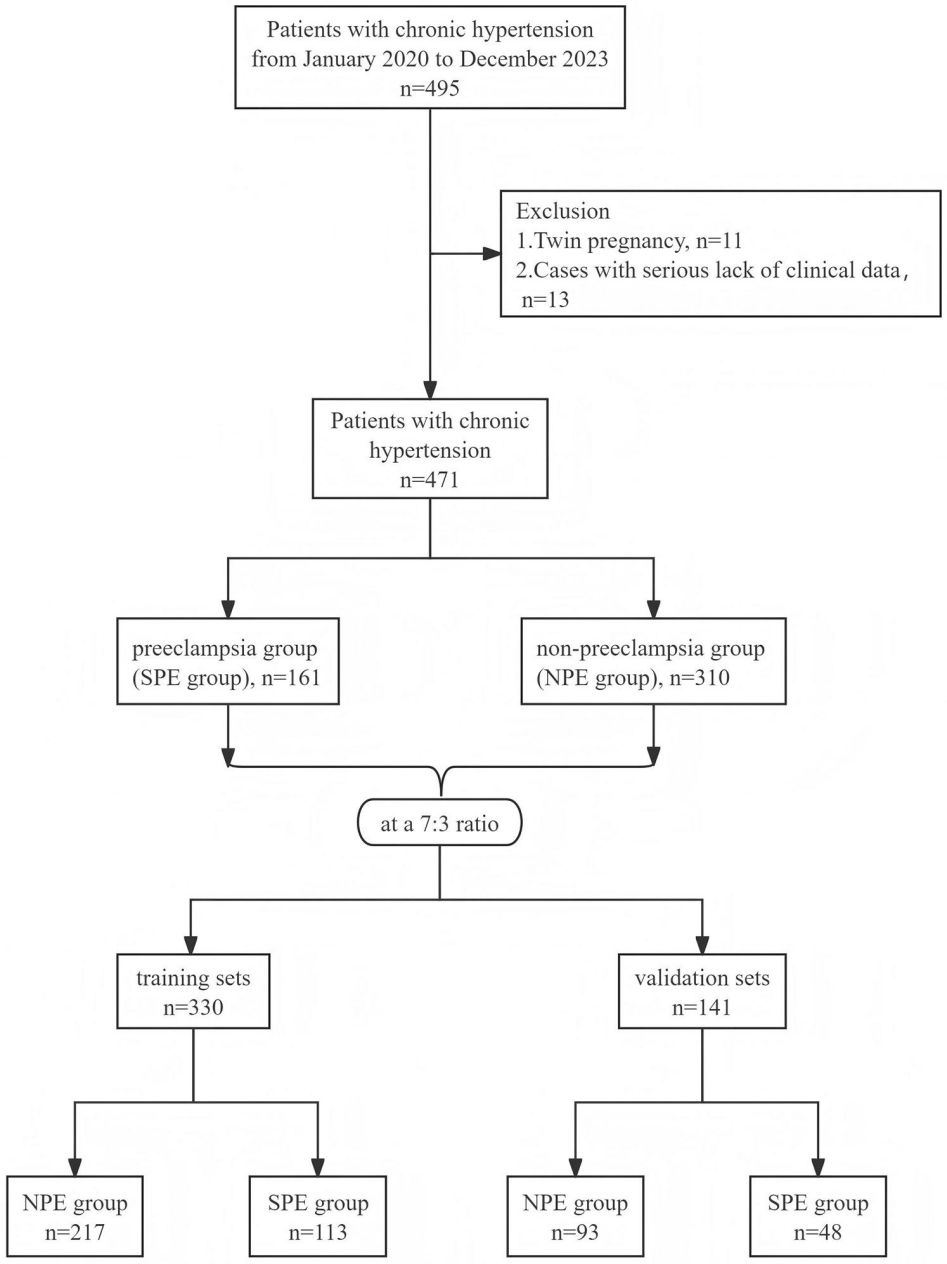
Flowchart of the screening process for the study population. SPE, superimposed pre-eclampsia; NPE, non-pre-eclampsia.

From January 2020 to December 2023, a total of 495 patients with CH were admitted to the Tongzhou Maternal and Child Health Care Hospital in Beijing. According to the inclusion and exclusion criteria, 24 cases (5.1% of screened patients) were excluded. In the end, a total of 471 patients with CH were included in this study and divided into two groups based on the presence or absence of PE: the superimposed PE group (SPE group, *n* = 161) and the non-PE group (NPE group, *n* = 310). The patients were randomly allocated into training and validation sets at a 7:3 ratio. The training set comprised 330 cases (NPE group: 217 cases [65.76%], SPE group: 113 cases [34.24%]), while the validation set consisted of 141 cases (NPE group: 93 cases [65.96%], SPE group: 48 cases [34.04%]) (Figure 1).

### Comparison of general information and clinical indicators

As can be noted from Tables 1–3, pre-pregnancy BMI, family history of hypertension, HGB, HCT, PDW, PT, PTA, TT, ALT, ALB, DBIL, GGT, GLU, BUN, UA, CK, TBA, HCY, GA, CysC were compared, and the differences were statistically significant.

**Table 1:** Comparison of general information in NPE and SPE group.

Item	NPE group (*n* = 217)	SPE group (*n* = 113)	*t*/*X*^2^	*P* value
Age (years)	31.53 ± 4.06	31.32 ± 5.10	0.390	0.697
Pre-pregnancy BMI (kg∕m^2^)	25.46 (23.23, 29.64)	27.34 (24.98, 31.24)	−3.087	0.002
Primipara [*n* (%)]	124 (57.14)	65 (57.52)	0.004	0.947
Natural pregnancy [*n* (%)]	207 (95.39)	112 (99.11)	2.146	0.143
History of chronic hypertension [*n* (%)]	33 (15.21)	15 (13.27)	0.223	0.636
History of diabetes [*n* (%)]	6 (2.76)	8 (7.08)	2.426	0.119
History of PCOS [*n* (%)]	7 (3.23)	6 (5.31)	0.391	0.532
History of PE [*n* (%)]	10 (4.61)	8 (7.08)	0.880	0.348
Family history of hypertension [*n* (%)]	21 (9.68)	20 (17.70)	4.394	0.036
SBP (mmHg)	140 (140, 144.0)	140 (140, 144.5)	−0.962	0.336
DBP (mmHg)	90 (90, 92)	90 (90, 90)	−0.591	0.555
Taking aspirin [*n* (%)]	115 (52.96)	60 (53.10)	0.000	0.986
Taking antihypertensive drugs [*n* (%)]	74 (34.10)	54 (47.69)	0.132	0.716
Number of prenatal examination (times)	15 (15, 15)	15 (14, 15)	−1.282	0.200
Gestational weight gain (kg)	11.38 ± 5.61	12.59 ± 6.36	−1.750	0.081
Hyperglycemia in pregnancy [*n* (%)]	111 (51.52)	52 (46.02)	0.784	0.376

BMI, body mass index; PCOS, polycystic ovary syndrome; PE, preeclampsia; SBP. systolic blood pressure; DBP, diastolic blood pressure.

**Table 2:** Comparison of blood system related clinical indicators in NPE and SPE group.

Item	NPE group (*n* = 217)	SPE group (*n* = 113)	*t*/*X*^2^	*P* value
HGB (g/L)	125.43 ± 11.65	122.42 ± 11.43	2.244	0.026
HCT (%)	37.20 ± 3.17	36.32 ± 3.13	2.401	0.017
PLT (*10^9^/L)	222.93 ± 56.76	219.28 ± 53.38	0.564	0.573
MCHC (g/L)	337.07 ± 8.92	336.90 ± 10.30	0.153	0.879
PDW (fL)	13.00 (11.00, 16.00)	16.00 (12.00, 16.00)	−3.349	0.001
PT (sec)	10.30 (10.00, 10.70)	10.10 (9.80, 10.50)	−3.670	0.000
PTA (%)	111.02 ± 7.84	115.72 ± 10.75	−4.100	0.000
APTT (sec)	27.13 ± 1.93	27.079 ± 2.40	0.222	0.824
FIB (g/L)	4.44 (4.09, 4.82)	4.54 (4.14, 4.95)	−0.916	0.360
TT (sec)	13.16 ± 0.92	13.58 ± 1.22	−3.555	0.000
D-D (mg/FEU)	1.42 (1.10, 1.92)	1.397 (1.10, 2.02)	−0.015	0.988

HGB, hemoglobin; HCT, hematocrit; PLT, platelet; MCHC, mean erythrocyte hemoglobin concentration; PDW, platelet distribution width; PT, prothrombin time; PTA, prothrombin time activity; APTT, activated partial thromboplastin time; FIB, fibrinogen; TT, thrombin time; D-D, d-dimer.

**Table 3:** Comparison of biochemistry-related clinical indicators in NPE and SPE group.

Item	NPE group (*n* = 217)	SPE group (*n* = 113)	*t*/*X*^2^	*P* value
ALT (U/L)	9 (7, 12)	10 (8, 14)	−1.979	0.048
AST (U/L)	14 (11, 17)	15 (12, 19)	−1.945	0.052
ALB (g/L)	36.16 ± 2.83	34.54 ± 3.13	4.758	0.000
GLOB (g/L)	25.83 ± 2.90	25.62 ± 3.27	0.745	0.457
TBIL (μmol/L)	6.40 (4.80, 8.50)	6.60 (4.95, 7.95)	−0.354	0.723
DBIL (μmol/L)	2.40 (1.90, 2.90)	1.90 (1.60, 2.60)	−4.868	0.000
ALP (U/L)	124 (103, 160)	124 (104, 145)	−0.562	0.574
GGT (U/L)	10 (7, 14)	12 (9, 18)	−2.869	0.004
GLU (mmol/L)	4.55 (4.20, 4.96)	4.75 (4.41, 5.34)	−2.949	0.003
BUN (mmol/L)	3.07 (2.55, 3.68)	3.56 (2.88, 4.39)	−3.408	0.001
SCREA (mmol/L)	52 (45, 57)	51 (45, 59)	−0.271	0.786
UA (mmol/L)	308.31 ± 71.96	345.81 ± 92.80	−3.745	0.000
CA (mmol/L)	2.27 ± 0.11	2.25 ± 0.14	1.282	0.201
TC (mmol/L)	5.75 ± 1.01	5.92 ± 1.37	−1.195	0.234
TG (mmol/L)	3.29 (2.56, 4.26)	3.36 (2.64, 4.54)	−1.147	0.251
HDL-C (mmol/L)	1.74 ± 0.36	1.77 ± 0.44	−0.398	0.691
LDL-C (mmol/L)	3.08 ± 0.84	3.17 ± 1.02	−0.646	0.591
CK (U/L)	44 (31, 71)	53 (36, 82)	−2.376	0.018
LDH (U/L)	160 (146, 178)	163 (148, 187)	−1.907	0.057
CK-MB (ng/ml)	1.64 (1.18, 2.29)	1.71 (1.23, 2.57)	−1.086	0.278
TBA (μmol/L)	2.20 (1.60, 3.10)	2.70 (2.00, 4.10)	−3.218	0.001
HCY (μmol/L)	6.70 (5.85, 7.90)	7.70 (6.70, 9.20)	−3.775	0.000
GA (%)	12.21 ± 1.05	11.70 ± 1.48	2.650	0.009
CysC (mg/L)	1.07 (0.93, 1.26)	1.15 (1.00, 1.35)	−2.912	0.004

ALT, alanine aminotransferase; AST, aspartate aminotransferase; ALB, albumin; GLOB, globulin; TBIL, total bilirubin; DBIL, direct bilirubin; ALP, alkaline phosphatase; GGT, gamma-glutamyl transferase; GLU, glucose; BUN, blood urea nitrogen; CREA, creatinine; UA, uric acid; CA, calcium; TC, total cholesterol; TG, triglyceride; HDL-C, high density lipoprotein cholesterol; LDL-C, low density lipoprotein cholesterol; CK, creatine kinase; LDH, lactate dehydrogenase; CK-MB, creatine kinase isoenzyme; TBA, total bile acids; HCY, homocysteine; GA, glycated albumin; CysC, cystatin-C.

### Multifactorial analysis of PE

The statistically significant indicators in Tables 1–3 were analyzed by multiple-factor binary logistic regression, and the results showed that peripheral blood levels of DBIL, GGT, GLU, CysC and pre-pregnancy BMI were independent influences on the occurrence of PE (*P* < 0.05), as shown in Table 4.

**Table 4:** Binary logistic regression analysis of PE in patients.

Variable	*β*	SE	Wald *X*^2^	*df*	*P* value	OR (95%CI)
Pre-pregnancy BMI	0.107	0.041	6.635	1	0.008	1.113 (1.028, 1.205)
DBIL	−1.012	0.276	13.410	1	0.000	0.364 (0.212, 0.625)
GGT	0.062	0.022	7.772	1	0.005	1.064 (1.019, 1.112)
GLU	0.409	0.176	5.386	1	0.020	1.505 (1.066, 2.127)
CysC	2.955	0.805	13.485	1	0.000	19.196 (3.966, 92.916)
Constant	−7.200	1.873	14.781	1	0.000	0.001

MI, body mass index; DBIL, direct bilirubin; GGT, gamma-glutamyl transferase; GLU, glucose; CysC, cystatin-C.

DBIL, Low DBIL reflects impaired antioxidant capacity, increasing oxidative stress and endothelial damage (13, 15).

GGT, Elevated GGT indicates hepatic ischemia and systemic inflammation, promoting vasoconstriction (16–18).

GLU, Hyperglycemia exacerbates insulin resistance and vascular smooth muscle proliferation, elevating hypertension risk (22, 23).

CysC, Increased CysC signals early glomerular dysfunction and renal arteriolar spasm in PE (20, 21).

BMI, High BMI drives chronic inflammation and cytokine release, accelerating endothelial injury (24, 25).

The regression equation is logit (*P*) = − 7.200 + 1.113 × Pre-pregnancy BMI − 0.364 × DBIL + 1.064 × GGT + 1.505 × GLU + 19.196 × CysC.

### Predictive value of each indicator for PE

**Figure 2:**
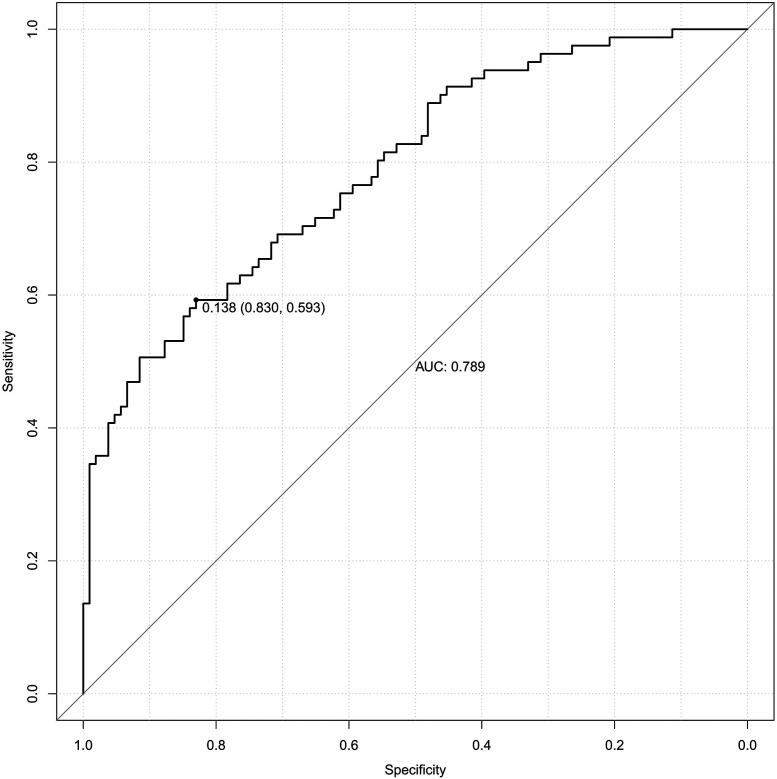
The receiver operating characteristic (ROC) curve of the combined diagnostic curve of DBIL, GGT, GLU, CysC and pre-pregnancy BMI. DBIL, direct bilirubin; GGT, gamma-glutamyl transferase; GLU, glucose; CysC, cystatin-C; BMI, body mass index; AUC, area under the curve.

Table 5 presents the ROC analysis of predictive performance of pre-pregnancy BMI, DBIL, GGT, GLU, CysC, and the combined predictive value for PE. The results showed that the area under the curve of the combined diagnostic curve of DBIL, GGT, GLU, CysC and pre-pregnancy BMI was 0.789 (Figure 2), the sensitivity was 0.593, and the specificity was 0.830, which was the largest area under the curve of the combined diagnostic curve compared to that of a single index, and it had a good clinical predictive value.

**Table 5:** Predictive performance of individual indicator and combined model for PE (ROC analysis).

Variable	threshold value	AUC	95%CI	Sensitivities	Specificities	Youden index	S.E.	*P*
Pre-pregnancy BMI	25.959	0.604	0.541, 0.666	0.655	0.539	0.194	0.032	0.002
DBIL	2.150	0.663	0.600, 0.726	0.637	0.637	0.274	0.032	0.000
GGT	16.500	0.622	0.541, 0.703	0.333	0.877	0.211	0.041	0.004
GLU	4.705	0.599	0.534, 0.664	0.540	0.651	0.191	0.033	0.003
CysC	1.310	0.598	0.533, 0.663	0.301	0.851	0.152	0.033	0.004
Joint prediction probability	0.535	0.789	0.725, 0.853	0.593	0.830	0.423	0.033	0.000

BMI, body mass index; DBIL, direct bilirubin; GGT, gamma-glutamyl transferase; GLU, glucose; CysC, cystatin-C; AUC, area under the curve; CI, confidence interval.

### Nomogram model for PE risk prediction

**Figure 3:**
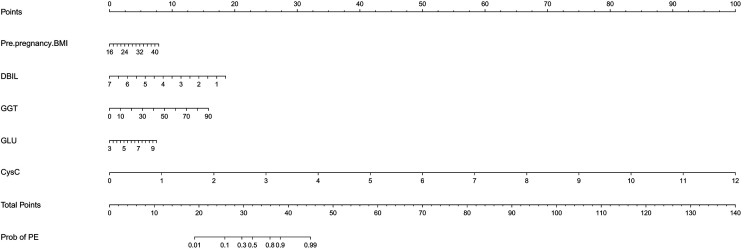
The nomogram model for PE risk prediction. BMI, body mass index; DBIL, direct bilirubin; GGT, gamma-glutamyl transferase; GLU, glucose; CysC, cystatin-C.

The independent factors screened by the multifactor logistic regression analysis were incorporated into the nomogram model. In the model, a score was obtained by positioning each predictor on the scale, and the overall score for each predictor was positioned on the total score axis, and corresponding risk coefficients reflect the patient's risk of PE, as shown in Figure 3.

### Model validation

**Figure 4:**
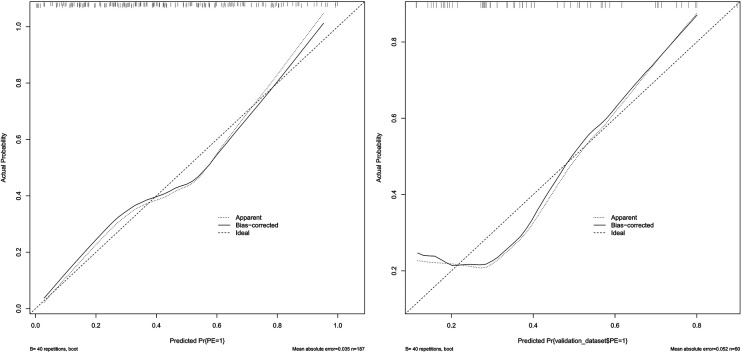
The calibration curve of the training set and validation set. (Solid line: observed risk; Dashed line: ideal calibration).

**Figure 5:**
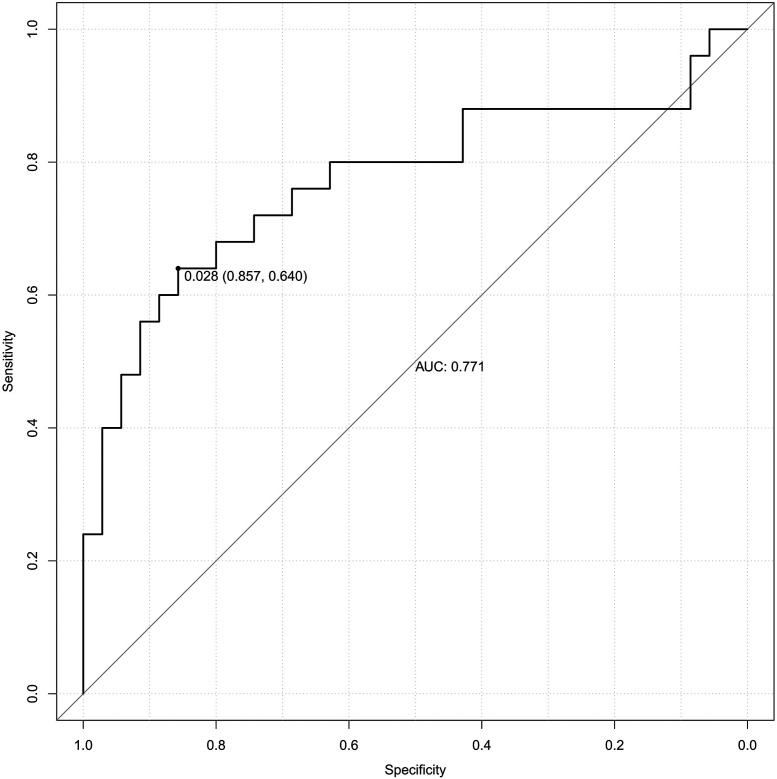
The receiver operating characteristic (ROC) curve of validation set. AUC, area under the curve.

The model was subjected to 1,000 bootstrap resampling, and the C-index was 0.789. The calibration curve (solid line in Figure 4) fluctuated around the ideal curve (dashed line in Figure 4), indicating good predictive performance and reproducibility of the model. The prediction factors pre-pregnancy BMI, DBIL, GGT, GLU, CysC in the validation dataset were entered into the prediction model to calculate the predicted probability, and the ROC curve was plotted based on the predicted probability (Figure 5). The AUC was 0.771. The results indicate that the prediction model has good predictive ability when used in validation data and has good portability.

### Decision curve

**Figure 6:**
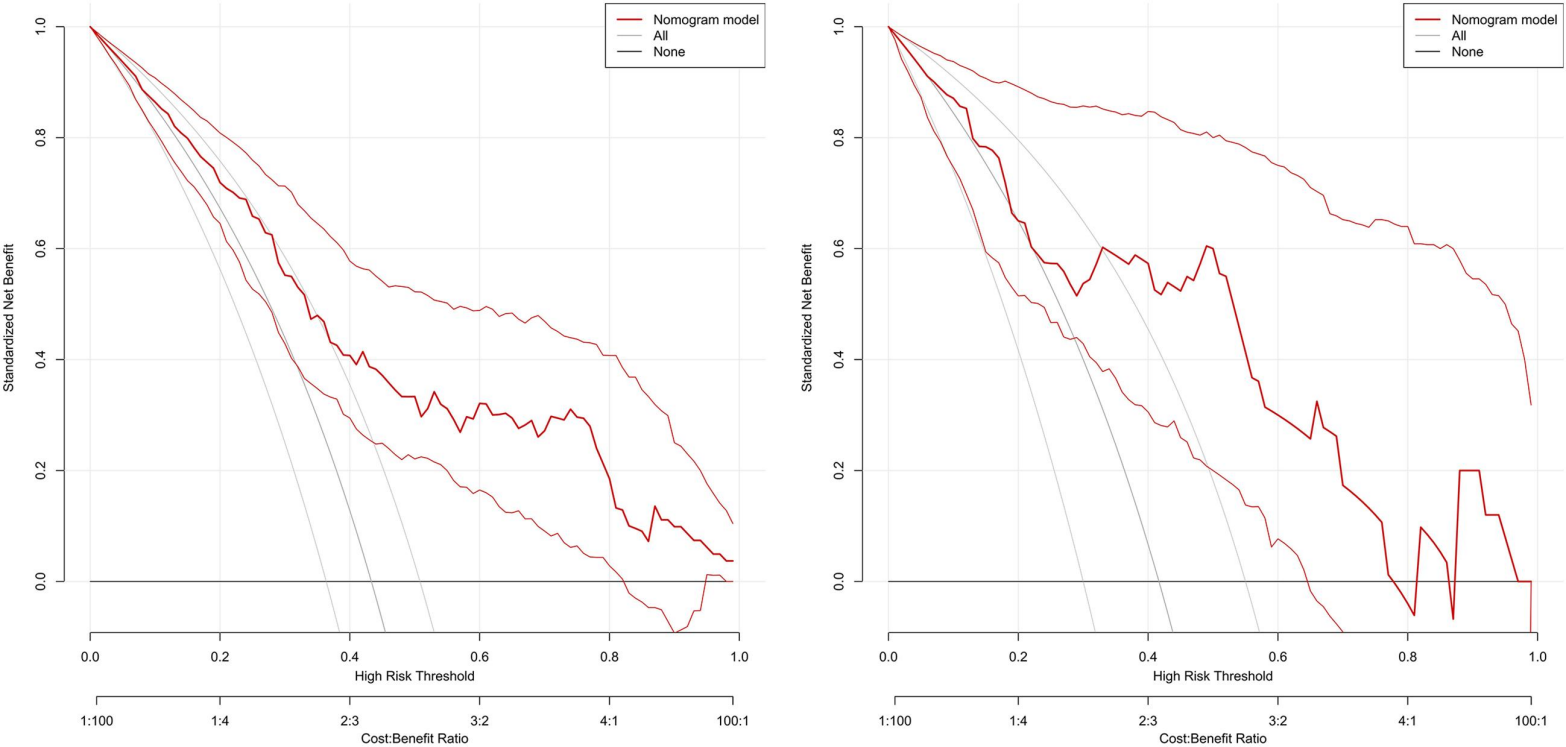
Clinical decision analysis curve of the training set and validation set.

The DCA curve showed that the use of this model for predicting the development of PE would be beneficial when the probability of occurrence was between 0.28 and 0.78, see Figure 6. DCA, decision curve analysis.

## Discussion

This study established a nomogram model for predicting PE risk in women with CH by integrating peripheral blood DBIL, GGT, GLU, CysC, and pre-pregnancy BMI (AUC = 0.789), demonstrating significantly superior comprehensive diagnostic efficacy compared to individual indicators. These parameters may reflect the multisystem pathogenesis of PE, including hepatorenal dysfunction, glucose metabolism disorders, and inflammation associated with overweight/obesity. This nomogram model targets primary care and resource-limited settings, utilizing routine laboratory parameters instead of specialized biomarkers like the sFlt-1/PlGF ratio or Doppler ultrasound (7, 8), which require skilled operators. Although the sFlt-1/PlGF ratio offers higher sensitivity (AUC = 0.83, 95% CI: 0.77–0.88) (7), our model presents a cost-effective alternative for initial PE risk stratification in CH patients.

PE is a pregnancy-specific disease that can cause maternal and peripartum infant death. The study (9) found that the average gestational age when blood pressure starts to rise in PE patients is 32.54 weeks, and the average gestational age at pregnancy termination is 33.92 weeks. This indicates that PE progresses rapidly, with only 7–10 days from the onset of elevated blood pressure to uncontrollable disease and pregnancy termination. Doctors often use dexamethasone to promote fetal lung maturation, reducing neonatal mortality, a treatment that takes 36 h. Therefore, there is an urgent need for a predictive method for early PE detection in clinical practice to allow for early intervention and ensure maternal and infant safety. Currently, several markers are used in clinical practice for predicting PE, including maternal age, body weight, primiparity, family history, chronic diseases, and pre-pregnancy hypertension (6, 10). In addition, metabolic markers such as mean platelet volume, triglycerides, lactate dehydrogenase, blood urea nitrogen, and fasting blood glucose levels are also considered (11). However, none of these markers exhibit high specificity. Biomarkers and imaging indicators, such as the sFlt-1/PIGF ratio and uterine artery pulsatility index, are significant in the prediction of PE (12). However, the markers involved are complex, expensive, and not suitable for large-scale clinical screenings. CH with superimposed PE represents a more severe form of hypertensive disorders during pregnancy. Following the rise in the number of older pregnant women, there has been an annual increase in the incidence of CH among pregnant women. However, research on this topic remains limited. Therefore, our study integrates commonly used, cost-effective and easily accessible clinical indicators to predict the risk of PE in pregnant women with CH.

PE often causes damage to target organs, including the liver and kidneys. Our study revealed an inverse relationship between decreased DBIL levels and the risk of PE, with significantly lower DBIL levels in the SPE group compared to the NPE group (*P* < 0.05). Bilirubin is typically regarded as a deleterious compound to the human body, it is predominantly utilized in clinical contexts to facilitate the diagnosis of hemolytic and hepatic disorders. However, recent studies have increasingly confirmed bilirubin's antioxidant capabilities (13), suggesting that low bilirubin levels elevate the risk of PE, potentially through several mechanisms: its antioxidant properties neutralize oxygen free radicals, thereby safeguarding vascular endothelial cells from oxidative stress-induced damage; it inhibits the formation of oxidized low-density lipoprotein, thereby preventing vascular inflammatory injury (14); and it suppresses the oxidation of low-density lipoprotein cholesterol, thereby maintaining redox balance and mitigating vascular endothelial damage (15). Our study revealed that the DBIL levels were significantly lower in the SPE group as compared to the NPE group. Despite being a prevalent laboratory indicator, DBIL is frequently disregarded by clinicians. Our study highlights the importance of DBIL in the early prediction of PE. Further foundational research is needed to explore and validate the underlying mechanisms.

Besides, our study has identified GGT as a predictive marker for PE, and our findings are consistent with those of Zhang et al. (16). Abnormal liver function during pregnancy is recognized as a significant risk factor for gestational hypertension and PE (17). Vascular injury is currently a recognized trigger for PE. Damage to vascular endothelium leads to elevated endothelin levels, and the increased endothelin entering the liver through the circulatory system induces hepatic microcirculatory contraction, thereby creating an ischemic and hypoxic environment. This results in hepatocyte damage and elevated ALT and AST levels (18, 19). ALT and AST can partially reflect the degree of vascular endothelial injury and exhibit a positive correlation with the occurrence of PE (20). Our research indicates that clinicians should place greater emphasis on GGT levels in conjunction with traditional liver function tests such as ALT and AST. This approach may enhance the monitoring of pregnancies in high-risk CH patients who are predisposed to developing PE.

The kidney is one of the main organs affected by PE. Our study suggest that elevated CysC (OR = 19.196) strongly indicates renal impairment, possibly due to decreased glomerular filtration rate and PE-related renal arteriolar spasm. PE patients exhibit significant continuous functional and structural changes in the kidneys, and the elevation of CysC may result from increased immune function and nucleated cell count in the body (21), aligning with Wen's proposal that CysC serves as an early predictive biomarker for PE (22). Regarding renal function indicators, most clinicians focus on uric acid, creatinine, and blood urea nitrogen, often overlooking CysC. Our results demonstrate that CysC can predict PE, suggesting that doctor should pay more attention to it in clinical practice. Although CysC demonstrated a strong association with PE (OR = 19.196), the wide confidence interval suggests variability in its effect size. This may reflect biological heterogeneity or sample size limitations, warranting cautious interpretation until validated in larger studies.

PE is significantly correlated with blood glucose levels and glycemic control during pregnancy (23).Our research found that hyperglycemia exacerbates the occurrence of PE. Hyperglycemia synergistically exacerbates hypertension through mechanisms involving insulin resistance and the proliferation of vascular smooth muscle, corroborating the findings of Cai et al. in patients with gestational diabetes (24). Elevated blood glucose levels result in insulin accumulation and persistent proliferation of vascular smooth muscle, which in turn leads to vascular spasms, increased vascular resistance, hypertension, and ultimately the development of PE (23). It is imperative to monitor blood glucose levels in pregnant women diagnosed with CH. In cases where elevated blood glucose is observed, heightened vigilance is required due to the increased risk of PE.

Research has demonstrated that oxidative stress and systemic inflammatory responses are pivotal factors in the pathogenesis of PE. Adiposity, encompassing both overweight and obesity, has been identified as a catalyst for chronic inflammatory conditions, which subsequently elevate levels of inflammatory cytokines. These cytokines further provoke a systemic inflammatory response within the vasculature, expediting the deterioration of vascular endothelial cells and culminating in the onset of PE (25, 26). We found that overweight or obesity (defined as a pre-pregnancy BMI of ≥25.96 kg/m^2^ as a risk threshold) can exacerbate PE, which is consistent with the aforementioned conclusions. This suggests that, within clinical practice, considerable emphasis should be placed on pre-pregnancy BMI. Providing effective guidance to women with CH for weight management prior to conception may contribute to a reduction in the incidence of PE.

The nomogram prediction model is the most widely applied among various predictive models and demonstrates significant advantages (27). International scholars suggest that nomogram models can visually illustrate the impact of different pre-pregnancy BMI levels and serum marker concentrations on the risk of PE, thereby assisting clinicians in better assessing patients' risk stratification (28–30). The independent risk factors identified for PE include DBIL, GGT, GLU, CysC and pre-pregnancy BMI. The integration of these factors provides a more accurate prediction of PE compared to the use of any single indicator. If a clinician identifies a patient with CH, she can assess DBIL, GGT, GLU, CysC, and pre-pregnancy BMI to estimate the likelihood of progression to PE. This approach can assist clinicians in optimizing pregnancy monitoring for CH patients who are at an elevated risk of developing PE. Early detection of disease progression enables the initiation of timely interventions, such as antihypertensive therapy and fetal lung maturation treatments, thereby potentially improving outcomes for both the mother and the infant.

Notably, over 50% of patients in both groups received aspirin prophylaxis, yet its use did not significantly differ between SPE and NPE groups (*P* = 0.986). This suggests that while aspirin is widely administered for PE prevention in high-risk pregnancies, its effect on mitigating superimposed PE in CH patients requires further investigation.

### Limitations

The model of this study was constructed based on data from a single center, with a relatively limited sample size, which may result in lower sensitivity (0.593) of the model. In the future, as multi-center data (e.g., multi-center) is included and emerging PE biomarkers (e.g., sFlt-1/PIGF) are integrated, the sensitivity of the model is expected to improve further. However, the model's specificity (0.830) and area under the curve (0.789) suggest a moderate predictive capacity, effectively minimizing false positives and rendering it suitable for preliminary screening in high-risk populations. Despite this, its generalizability may be constrained by the single-center retrospective design. Additionally, we recommend prospective validation in future studies, incorporating machine learning to optimize variable weights, and conducting external validation in different populations to further evaluate the stability and clinical application value of the model.

## Conclusion

The nomogram model developed in this study offers an economically efficient and highly accessible tool for the risk stratification management of PE in women with CH. It is particularly well-suited for primary healthcare settings with limited resources. The clinical translation of this model has the potential to enhance maternal and neonatal outcomes in resource-constrained regions. While further refinement is necessary, the model's reliance on routine clinical indicators is consistent with the principle of “accessibility prioritization” in precision medicine. Future applications could improve its effectiveness through technological integration, such as the use of mobile health platforms.

## Data Availability

The raw data supporting the conclusions of this article will be made available by the authors, without undue reservation.
